# Numerical modeling of mosquito population dynamics of *Aedes aegypti*

**DOI:** 10.1186/s13071-018-2829-1

**Published:** 2018-04-16

**Authors:** William M. S. Yamashita, Shyam S. Das, Grigori Chapiro

**Affiliations:** 1 Graduate Program in Computational Modeling, Federal University of Juiz de Fora, Juiz de Fora, MG 36036-330 Brazil; 2 Department of Mathematics, Federal University of Juiz de Fora, Juiz de Fora, MG 36036-330 Brazil

**Keywords:** *Aedes aegypti*, Partial differential equations, Finite volume method

## Abstract

**Background:**

The global incidences of dengue virus have increased the interest in studying and understanding the mosquito population dynamics. It is predominantly spread by *Aedes aegypti* in the tropical and sub-tropical countries in the world. Understanding these dynamics is important for public health in countries where climatic and environmental conditions are favorable for the propagation of these diseases. For this reason, a new model has been proposed to investigate the population dynamics of mosquitoes in a city.

**Methods:**

The present paper discusses the numerical modeling of population dynamics of *Ae. aegypti* mosquitoes in an urban neighborhood of a city using the finite volume method. The model describes how populations spread through the city assisted by the wind. This model allows incorporating external factors (wind and chemical insecticides) and topography data (streets, building blocks, parks, forests and beach). The proposed model has been successfully tested in examples involving two Brazilian cities (City center, Juiz de Fora and Copacabana Beach, Rio de Janeiro).

**Results:**

Invasion phenomena of *Ae. aegypti* mosquitoes have been observed in each of the simulations. It was observed that, inside the blocks, the growth of the population for both winged and aquatic phase causes an infestation of *Ae. aegypti* in a short time. Within the blocks the mosquito population was concentrated and diffused slowly. In the streets, there was a long-distance spread, which was influenced by wind and diffusion with a low concentration of mosquito population. The model was also tested taking into account chemical insecticides spread in two different configurations. It has been observed that the insecticides have a significant effect on the mosquito population for both winged and aquatic phases when the chemical insecticides spread more uniformly along all the streets in a neighborhood of a city.

**Conclusions:**

The presented methodology can be employed to evaluate and to understand the epidemic risks in a specific region of the city. Moreover the model allows an increase in efficiency of the existing mosquito population control techniques and to theoretically test new methods before involving the human population.

**Electronic supplementary material:**

The online version of this article (10.1186/s13071-018-2829-1) contains supplementary material, which is available to authorized users.

## Background

Dengue is considered as one of the major public health problems by the World Health Organization (WHO) in the world [1]. It is the most rapidly spreading vector-borne disease in the world [2] with nearly 400 million people infected each year and an estimated 25,000 deaths; this leads to an enormous economic cost in affected countries, comparable to that of malaria [3]. In the last 50 years this endemic disease has increased 30-fold with increasing geographical expansion to new countries, and in the present decade, from urban to rural settings [2]. This upward trend is due to increases in long-distance travel, population growth, urbanization, lack of sanitation and ineffective mosquito control [4]. Dengue is a serious health problem in Brazil due to a favorable climate and environmental conditions for the proliferation of *Ae. aegypti* mosquito populations [5]. It has been reported that 70% of dengue cases in Latin America and Caribbean countries occurred only in Brazil (from 1992 to 2002), where the number of cases has increased greatly in recent years [6]. Besides dengue, Zika virus has emerged as one of the most challenging threats to human health [3]. It has spread rapidly throughout the Americas and beyond since 2015, causing birth defects in children of infected mothers. Chikungunya and yellow fever are both painful and debilitating diseases which can prove fatal and have both experienced epidemics in recent years [3].

The *Ae. aegypti* mosquito is the primary vector for spreading viral diseases such as dengue, Zika, yellow fever and chikungunya, and every year hundreds of millions of humans are affected [3, 4, 7]. The dispersion of *Ae. aegypti* has increased in the urban areas of the planet. This species proliferates in close proximity to human communities using artificial water deposits as the breeding place [8]. For simplicity, in the rest of the text, we only focus on dengue.

Previous studies address different strategies to control the population of *Ae. aegypti*, for example: using bio-insecticide, larvae-eating fish species, and chemical insecticides [1]; through controlling the breeding of mosquitoes in home environment during the year [9]; or using genetically modified mosquitoes [10].

Any feasible public policy for controlling the dengue epidemics in tropical climates must necessarily include appropriate strategies for minimizing the mosquito population factor [5]. Studying the mosquitoes’ propagation has important implications for understanding the hyperendemicity patterns of mentioned diseases and also facilitating the design and development of the vaccination strategies [11].

There are several strategies in modeling mosquito populations in the literature. For example, the mathematical model based on ordinary differential equations (ODEs) was used in [12] to study the importance of the temperature and precipitation on the patterns of mosquito population. Malik at al. [13] used Graph Theory to extend ODE analysis to two dimensions. This approach presents difficulties due to mosquitoes’ erratic movement that is similar to the diffusion process. Some authors [5, 14–17] took diffusion into account and studied one-dimensional spatial population dynamics of *Ae. aegypti* using partial differential equations (PDEs) describing the life-cycle of the mosquito *Ae. aegypti*. Maidana et al. [14] studied solutions in the form of traveling waves corresponding to mosquito *Ae. aegypti* invasion processes. The rate of spread of the disease was determined by the application of traveling wave solutions for the corresponding system of PDEs. Takahashi et al. [5] showed the existence of traveling wave solutions in many different situations. A numerical study was carried out to relate the speed of wave fronts and different crucial parameters associated with the dengue modeling. This approach was further extended in [16, 17], where a rigorous analysis of the model proposed in [5] was performed. However, one-dimensional models do not support realistic scenarios like topography.

There are few works in the literature addressing two-dimensional realistic modeling for modern cities. For example, the spread of the mosquito *Ae. albopictus* was modeled taking into account the environmental parameters such as wind, temperature and landscape [18]. The authors also investigated the use of the sterile insect technique (SIT), which introduces a large number of sterile male insects in the environment. Since this model is based on a behavior approach it leads to a large system of PDEs, which is difficult to solve numerically.

A rigorous mathematical analysis was carried out in [19] for the system of nonlinear partial differential equations corresponding to a generalization of a mathematical model for geographical spreading of dengue disease proposed in [14]. It is interesting to notice that this purely mathematical model allows higher dimensions and includes one term, which can model the abiotic effects such as variations in temperature, humidity and wind velocity.

In the present work, we followed Takahashi et al. [5] and Maidana & Yang [14] and studied the spatial population dynamics of *Ae. aegypti* using a mathematical model based on a system of reaction-convection-diffusion PDEs. This model allows incorporating external factors (wind and chemical insecticides) and topography data (streets, building blocks, parks, forests and beach).

Two-dimensional numerical simulations were carried out using a finite volume method (FVM) on the dispersion model for mosquito population. Initially, the spreading of *Ae. aegypti* mosquitoes in an generic city quarter (or apartment complex) was examined. Then, we investigated the population dynamics of *Ae. aegypti* mosquitoes in a dense neighborhood of the city Juiz de Fora. In order to control the population of mosquitoes, chemical insecticides were used in the model. Furthermore, we investigated the population dynamics of *Ae. aegypti* mosquitoes in the city of Rio de Janeiro. In each of the simulations, biological invasion of *Ae. aegypti* mosquitoes was studied.

The presented model is a tool that can be used to predict mosquitoes’ propagation in a city. It offers new possibilities to control the vector propagated diseases.

## Methods

### Studied areas

**Fig. 1:**
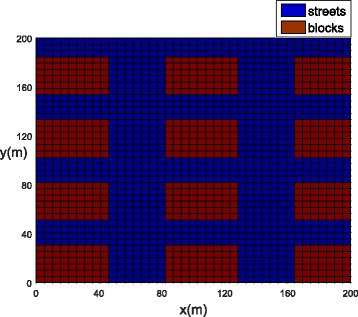
Example 1: computational domain for functions *ψ*, *v*_*x*_, *v*_*y*_, *D*_*x*_, *D*_*y*_, *h*_1_, and *h*_2_, including blocks (red) and streets (blue). The values are given in Table 2

Three different areas were considered in the present study. Example 1 represents the generic case containing only streets and building blocks (for example, apartment complexes) (Fig. 1). It was used to show the general behavior of the model and to test the algorithm. The size of the studied area, in this case, was 200 × 200 m.

**Fig. 2:**
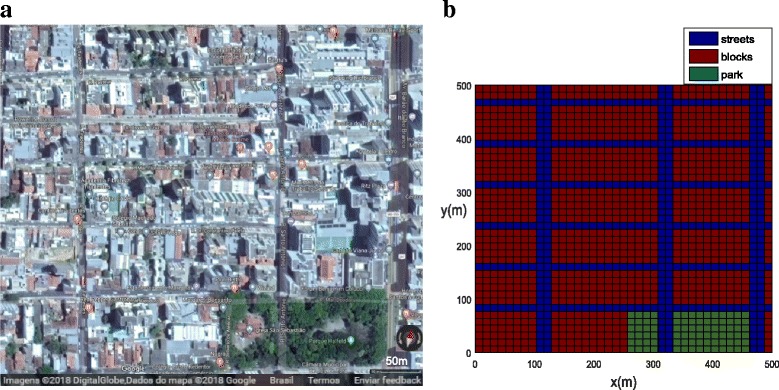
Examples 2 and 3: the small neighborhood in the city of Juiz de Fora (Park Halfeld). **a** Google Maps image of Juiz de Fora center. **b** Computational domain for functions *ψ*, *v*_*x*_, *v*_*y*_, *D*_*x*_, *D*_*y*_, *h*_1_, and *h*_2_, including blocks (red), park (green) and streets (blue). The values are given in Table 3

Examples 2 and 3 investigated the population dynamics of *Ae. aegypti* mosquitoes in the busy neighborhood in the city center of Juiz de Fora, located in south-eastern Brazilian state Minas Gerais (see Fig. 2a and Additional file 1: Figure S1). Its humid subtropical climate is favorable for the proliferation of *Ae. aegypti* mosquitoes. In 2016, 528,441 probable cases and incidences of dengue, 1431 probable cases and incidences of chikungunya fever and 14,436 probable cases and incidences of Zika virus fever were reported in the State of Minas Gerais [20]. In the city of Juiz de Fora 19,746 dengue cases were reported during 2016 [21]. The neighborhood in Fig. 2a contains streets, 17 building blocks and one park (Park Halfeld, indicated in green in Fig. 2b). The neighborhood is represented mathematically in Fig. 2b with a size 500 × 500 m.

**Fig. 3:**
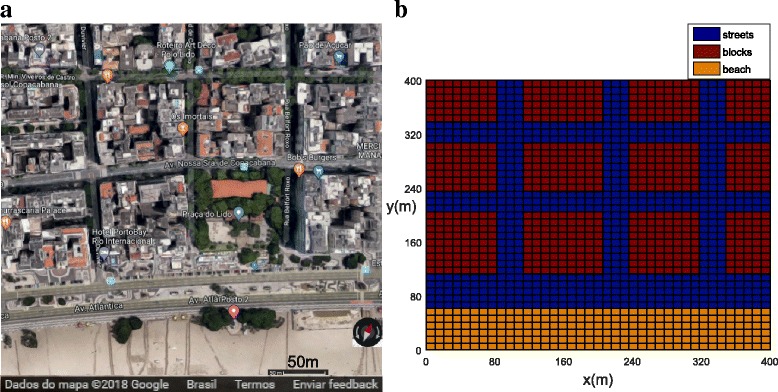
Example 4: the small neighborhood in Copacabana Beach, Rio de Janeiro city. **a** Google Maps image of Copacabana Beach. **b** Computational domain for functions *ψ*, *v*_*x*_, *v*_*y*_, *D*_*x*_, *D*_*y*_, *h*_1_, and *h*_2_ for Rio de Janeiro, including blocks (red), beach (orange) and streets (blue). The values are given in Tables 6 and 7

Example 4 considers a busy neighborhood (Copacabana Beach) in the city of Rio de Janeiro (see Fig. 3a and Additional file 2: Figure S2). It is the second largest city in Brazil and the sixth most populous city in the Americas. It is located in the south-eastern Brazilian State of Rio de Janeiro. The weather of Rio de Janeiro city is tropical monsoon and is characterized by a long period of heavy rain between December and March. The mixture of hot summer and heavy rain creates ideal conditions for the proliferation of *Ae. aegypti* mosquitoes. Rio de Janeiro has become one of the most endemic cities in Brazil with a long history of dengue virus circulation. In 2016, 85,200 probable cases and incidences of dengue, 17,888 probable cases and incidences of chikungunya fever and 68,542 probable cases and incidence of Zika virus were reported in the state of Rio de Janeiro [20]. For the city of Rio de Janeiro, 25,837 dengue cases were reported in 2016 alone [22]. The neighborhood from Fig. 3a containing streets, buildings and beach is represented mathematically in Fig. 3b with a size of 400 × 400 m.

The main difference between Example 4 and Example 3 is the presence of beach jointly with the periodic winds affecting the dispersion of the mosquito population. In order to study this phenomenon the vegetation areas are neglected.

The mathematical domain Ω represents a neighborhood of a city. For simplicity, all the examples presented in this paper consider the domain divided into rectangular blocks, which represent different parts of the city, such as building, gardens, squares, streets, beach, forests, etc. The difference between all these blocks is the mosquito population support, displacement and mortality coefficients. This approach allows us to model and simulate the population dynamics with more precision.

Notice that the incidence of dengue and mosquito population is known for quarters and not for city blocks. That is why, while it is important to model areas with abundant vegetation, few vegetation areas can be regarded as homogeneous with the rest of the quarter. To obtain the parameter values for the city blocks, the satellite images can be employed through color analysis. However, it has not been used in the present work, and left for future studies.

### Application of insecticides

During the periods of higher incidence of *Ae. aegypti* mosquitoes, which corresponds to three summer months in studied areas, strong measures are required to control the vector populations and the transmitted diseases. Applications of space sprays are an important means of reducing *Ae. aegypti* populations. Space sprays include ultra-low volume aerosols, thermal fogs, or dusts using truck-mounted equipment. Adulticiding applications exert little or no effect on the aquatic phases of *Ae. aegypti*, and adults will continue to emerge after spraying [23].

**Fig. 4:**
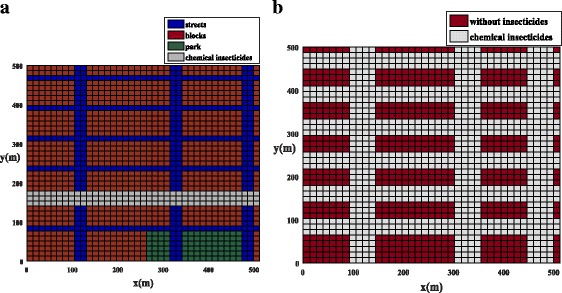
Example 3: computational domain for functions *h*_1_, and *h*_2_ for Juiz de Fora, where the chemical insecticide is represented by the grey color. **a** One street with chemical insecticides. The figure represents the values of the parameters of Tables 3 and 4. **b** All streets with chemical insecticides. The figure represents the values of the parameters of Table 5

Therefore, in the present work, we only discuss the chemical control for the adult stage of the mosquito for the winged phase. In order to investigate the mosquito control strategies in a city, we have used two different ways of applying chemical insecticides in the neighborhood of Juiz de Fora. Initially, the insecticides were used along one street. As shown in Fig. 4a, one street with the surrounding area (grey color) has been selected for the use of insecticides to control the population of mosquitoes. Later, the effect of insecticides on mosquito population has been studied along all the streets of the city center in Juiz de Fora (Fig. 4b). Note that for both of the simulations we used the same amount of insecticides.

### Mathematical model

A deterministic mathematical model is proposed here to investigate the population dynamics of *Ae. aegypti* mosquitoes. The following model is based on the two-dimensional system of PDEs for population dynamics of *Ae. aegypti* mosquitoes. We are interested in an urban spatial scale, where diffusion represents the dispersion of the mosquitoes due to autonomous and random movements of the winged females. To simplify the vital biological dynamics of mosquitoes we follow [5, 16, 17] and consider two subpopulations: a winged and mobile form (mature female mosquitoes); and an aquatic and static form, which includes eggs, larvae and pupae. At each space point (*x*, *y*) and time *t*, the spatial density of the winged phase and aquatic phase are denoted by *M*(*x*, *y*, *t*) and *A*(*x*, *y*, *t*), respectively. The governing equations are:

1 $$ \frac{\partial \left(\psi M\right)}{\partial t}=-\nabla \cdot (vM)+\nabla \cdot \left(D\nabla M\right)+\gamma A\left(1-\frac{M}{k_1}\right)-\left({\mu}_1+{h}_1\right)M, $$

2 $$ \frac{\partial \left(\psi A\right)}{\partial t}=r\left(1-\frac{A}{k_2}\right)M-\left({\mu}_2+{h}_2+\gamma \right)A, $$

3 $$ M\left(\cdot, 0\right)={M}_0\left(\cdot \right), $$

4 $$ A\left(\cdot, 0\right)={A}_0\left(\cdot \right), $$

where *ψ*(*x*, *y*) represents the coefficient for mosquitoes area support explained below; *v*(*x*, *y*, *t*) is the wind velocity (ms^-1^); *D*(*x*, *y*) is the diffusion coefficient (m^2^s^-1^); *μ*_*i*_ is the mortality rate (day^-1^); *k*_*i*_ is the carrying capacity (day^-1^); *γ* is the specific rate of maturation of the aquatic phase into winged phase (day^-1^); *r* is the oviposition rate of the female mosquitoes (day^-1^); and *h*_*i*_(*x*, *y*, *t*) is the coefficient of the killing term (day^-1^), where index *i =* 1 corresponds to winged phase and *i =* 2 corresponds to aquatic phase.

The population of winged female *Ae. aegypti* mosquitoes has been modeled using Eq. (1). Since, the concentration of both the winged and the aquatic phase are not uniform in the city, we have introduced a mosquitoes area support coefficient *ψ* for both phases in Eqs. (1) and (2). It takes value from 0 to 1 and means how many mosquitoes can be supported in the area. Thus the total population of winged and aquatic phase is given by (*ψ*(*x*, *y*)*M*(*x*, *y*, *t*)) and (*ψ*(*x*, *y*)*A*(*x*, *y*, *t*)), respectively. The first term in Eq. (1) represents the rate of change of total population for the winged phase. The transport phenomenon of the population due to the wind is represented by the second (advection) term in Eq. (1) [5]. The dispersion of the mosquitoes was modeled using the third (diffusion) term in Eq. (1). The fourth term in Eq. (1) represents the coupling between the winged female phase and aquatic phase form. It describes the rate of maturation of the aquatic form into winged phase. This term depends on the function *k*_*1*_, which describes a carrying capacity related to the amount of available nutrients. The fifth term in Eq. (1) represents mosquito death by natural and induced causes. The population of the aquatic phase of *Ae. aegypti* mosquitoes was modeled using Eq. (2). The first term in Eq. (2) represents the rate of change of the total population for the aquatic phase. The rate of oviposition by female mosquitoes, which is the only source in aquatic form, is proportional to the total mosquito population in the winged phase. It is also regulated by a carrying capacity effect depending on the occupation of the available breeders: *rM*(*x*, *y*, *t*)(*1 − A*(*x*, *y*, *t*)*/k*_*2*_). The third term in Eq. (2) represents mosquito population death in aquatic phase by natural or induced causes, and maturation of the aquatic phase to winged phase. The term *γA* represents the number of mosquitoes changed from aquatic phase to winged phase. It reduces the mosquito population in the aquatic phase. Equations (3) and (4) represent the initial population of the winged and aquatic phase, respectively.

### Boundary conditions

In order to solve the system of PDEs, we need to specify the boundary conditions of the domain. The case of known net influx of mosquitoes to the city along one side of the domain is referred to mathematically as Neumann boundary conditions. For example, this is the case when considering an isolated part of the city with zero net influx of mosquitoes along each side of the domain. We consider *∂*_*x*_*M =* 0 at the left and right sides and *∂*_*y*_*M =* 0 on the top and bottom sides. This type of boundary is used in Examples 1, 2 and 3 in the Results section.

The case, when the amount of mosquitoes is known at one of the sides of the domain, is called Dirichlet boundary conditions. For example, when modeling sea coastal areas, the mosquito population was assumed to be zero along the side which faces the sea. This case is studied in Example 4 in the Results section, where zero Neumann boundary conditions have been specified along the other three sides of the domain.

### Numerical methods

**Fig. 5:**
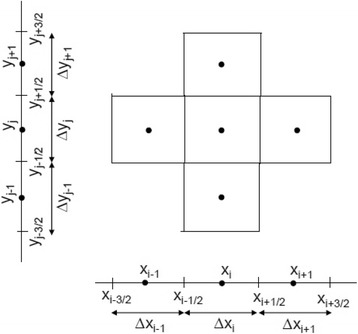
Representation of a “cell” (*x*_*i*_ , *y*_*j*_ ) following [25]

The governing equations describing the population dynamics of *Ae. aegypti* have been discretized using standard FVM, see [24–27] for details. The domain is given by *Ω = [0*, *L] × [0*, *L]*. The system (1)-(2) can be written as a scalar transport equation:

5 $$ \frac{\partial \left(\psi U\left(x,y,t\right)\right)}{\partial t}+\nabla \cdot \left( vU\left(x,y,t\right)\right)=\nabla \cdot \left(D\nabla U\left(x,y,t\right)\right)+\phi \left(U,x,y,t\right), $$

where *U = [M A]*^*T*^, *ϕ* is the source term and the others terms are the same as in Eq. (1). In order to solve the problem, we divided the domain *Ω* into “cells” or control volumes (see Fig. 5).

To obtain the discretization, we integrate the Eq. (5) in the cell centered in (*x*_*i*_, *y*_*j*_)*.* For the temporal derivative, it results in:

6 $$ {\int}_{y_{j-1/2}}^{y_{j+1/2}}{\int}_{x_{i-1/2}}^{x_{i+1/2}}\frac{\partial \left(\psi U\right)}{\partial t} dxdy\approx \Delta {x}_i\Delta {y}_j\frac{\psi_{i,j}^{n+1}{U}_{i,j}^{n+1}-{\psi}_{i,j}^n{U}_{i,j}^n}{\Delta \mathrm{t}}, $$

where *U*(*x*_*i*_, *y*_*j*_, *t*_*n*_) *= U*^*n*^_*i*,*j*_. The integral for the source term is

7 $$ {\int}_{y_{j-1/2}}^{y_{j+1/2}}{\int}_{x_{i-1/2}}^{x_{i+1/2}}\phi dxdy\approx \Delta {x}_i\Delta {y}_j{\phi}_{i,j}^n\cdot $$

For the convective and diffusion terms in Eq. (5) consider the derivative only in the *X* direction. The integral of flow term is:

8 $$ {\displaystyle \begin{array}{l}{\int}_{y_{j=1/2}}^{y_{j+1/2}}{\int}_{x_{i-1/2}}^{x_{i+1/2}}\frac{\partial }{\partial x}\left(-{v}_xM+\frac{\partial \left({D}_xM\right)}{\partial_x}\right) dxdy=\\ {}={\int}_{y_{j-1/2}}^{y_{j+1/2}}\left(\left[-{v}_xM+{D}_x\frac{\partial M}{\partial x}\right]\left|{}_{\left({x}_{i+1/2},{y}_j\right)}\right.-\left[-{v}_xM+{D}_x\frac{\partial M}{\partial x}\right]\left|{}_{\left({x}_{i-1/2},{y}_j\right)}\right.\right) dy\approx \\ {}\approx \Delta {y}_j\left[-{\left({v}_x\right)}_{\left({x}_{i+1/2},{y}_j\right)}{M}_{\left({x}_{i+1/2},{y}_j\right)}+{\left({D}_x\right)}_{\left({x}_{i+1/2},{y}_j\right)}\left(\frac{M_{i+1,j}-{M}_{i,j}}{\Delta {x}_i}\right)+\right.\\ {}\left.+{\left({v}_x\right)}_{\left({x}_{i-1/2},{y}_j\right)}{M}_{\left({x}_{i-1/2},{y}_j\right)}-{\left({D}_x\right)}_{\left({x}_{i-1/2},{y}_j\right)}\left(\frac{M_{i,j}-{M}_{i-1,j}}{\Delta {x}_i}\right)\right],\end{array}} $$

where *v =* ( *v*_*x*_, *v*_*y*_), *D =* (*D*_*x*_, *D*_*y*_). To find the values of ${M}_{\left({x}_{i+1/2},{y}_j\right)},{M}_{\left({x}_{i-1/2},{y}_j\right)},$ we must analyze the sign of ${\left({v}_x\right)}_{\left({x}_{i+1/2},{y}_j\right)}$ and ${\left({v}_x\right)}_{\left({x}_{i-1/2},{y}_j\right)}$. If ${\left({v}_x\right)}_{\left({x}_{i+1/2},{y}_j\right)}^n>0$, then ${M}_{\left({x}_{i+1/2},{y}_j\right)}^n\approx {M}_{i,j}^n$ and ${M}_{\left({x}_{i-1/2},{y}_j\right)}^n\approx {M}_{i-1,j}^n$. If ${\left({v}_x\right)}_{\left({x}_{i+1/2},{y}_j\right)}^n<0$, then ${M}_{\left({x}_{i+1/2},{y}_j\right)}^n\approx {M}_{i+1,j}^n$ and ${M}_{\left({x}_{i-1/2},{y}_j\right)}^n\approx {M}_{i,j}^n$. The derivation is analogous for the *Y* direction, see [25].

The System (1)-(4) was solved using an in-house finite volume code developed using MATLAB. The forward Euler method was used for time integration. For all time steps the Courant-Friedrichs-Lewy (CFL) condition has been tested guaranteeing the numerical stability.

## Results

In this section we present the numerical results obtained by solving System (1)-(4). The simulations were carried out for four different scenarios. The first example corresponds to a city quarter. In the second and third examples, we investigate the population dynamics of *Ae. aegypti* mosquitoes in the city of Juiz de Fora with and without the use of chemical insecticides. In the fourth example, we present the numerical results for Copacabana Beach in the city of Rio de Janeiro. The model parameters summarized in Table 1 have been taken from the literature [5]. All the simulations were performed for six weeks.

**Table 1:** Dimensional parameters used in all simulations [5]. Some parameter values vary from one simulation to another, these appear only as units in the table

Parameter	Description	Base value/units
*D*	Diffusion coefficient	1.25 × 10^-2^ m^2^s^-1^
*D*_*x*_	Diffusion coefficient (*X* direction)	(m^2^s^-1^)
*D*_*y*_	Diffusion coefficient (*Y* direction)	(m^2^s^-1^)
*γ*	Specific rate of maturation of the aquatic phase into winged phase	0.2 day^-1^
*r*	Oviposition rate of the female mosquitoes	30 day^-1^
*k*_*1*_	Carrying capacities of the winged phase	25 day^-1^
*k*_*2*_	Carrying capacity of the aquatic phase	100 day^-1^
*μ*_*1*_	Mortality rate of the winged phase	4.0 × 10^-2^ day^-1^
*μ*_*2*_	Mortality rate of the aquatic phase	1.0 × 10^-2^ day^-1^
*ν*	Wind velocity	5.0 × 10^-2^ ms^-1^
*ν*_*x*_	Wind velocity (*X* direction)	(ms^-1^)
*ν*_*y*_	Wind velocity (*Y* direction)	(ms^-1^)
*h*_*1*_	Coefficient of the killing term of the winged phase	0 day^-1^
*h*_*2*_	Coefficient of the killing term of the aquatic phase	0 day^-1^
*ψ*	Coefficient for mosquitoes’ area support	[ . ]

### Example 1: Generic blocks

Let us consider an arbitrary urban square neighborhood composed of building blocks (red) and streets (blue) as plotted in Fig. 1. The model parameters are presented in Table 2. The component of the wind velocity in the *Y* direction is assumed to be larger than the component in the *X* direction.

**Table 2:** Dimensional parameter values used for Example 1

Parameter	Blocks	Streets
*ψ*	1	0.3
*ν*_*x*_	0	5.0 × 10^-2^
*ν*_*y*_	0	1.5 × 10^-1^
*D*_*x*_	3.75 × 10^-3^	1.25 × 10^-2^
*D*_*y*_	3.75 × 10^-3^	1.25 × 10^-2^

**Fig. 6:**
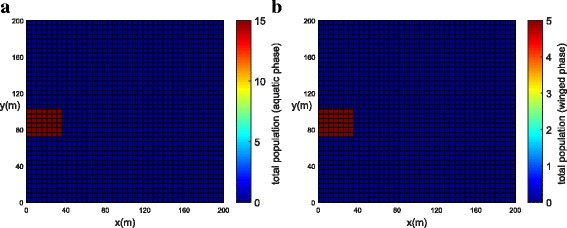
Example 1: initial condition for both populations (*t* = 0). **a** Aquatic phase. **b** Winged phase

**Fig. 7:**
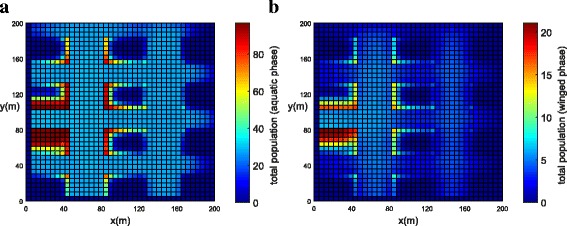
Example 1: population propagation inside general blocks (*t* = 21 days). **a** Total population for aquatic phase (*ψ × A*). **b** Total population for winged phase (*ψ × M* )

**Fig. 8:**
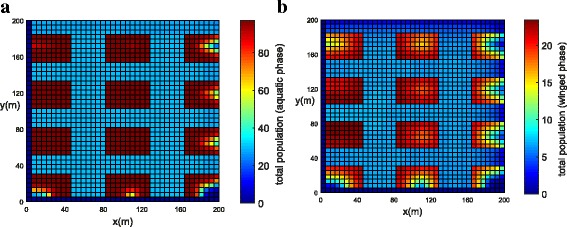
Example 1: population propagation inside general blocks (*t* = 42 days). **a** Total population for aquatic phase (*ψ × A*). **b** Total population for winged phase (*ψ × M* )

The population concentrations for both phases (winged and aquatic) are shown for different times in Fig. 6a, b (*t =* 0), Fig. 7a, b (*t =* 21 days) and Fig. 8a, b (*t =* 42 days). Notice that the population for both phases slowly penetrates, by diffusion, from the street into blocks as shown in Fig. 7a, b. The population for both phases spreads rapidly in the streets due to the presence of wind and larger value of diffusion coefficient when compared to the blocks. The population of mosquitoes for both phases spreads quickly along the *Y* direction explained by the presence of larger component of wind velocity along the *Y* direction. As time progresses, we observe that the population of mosquitoes increases inside the blocks (Fig. 8a, b). The increase in population could be explained by the invasion of mosquitoes into the blocks. Furthermore, it is observed that the invasion process decreases inside the blocks closer to the boundary due to zero influx of mosquitoes near the boundary.

### Example 2: City center of Juiz de Fora

In order to simulate the population dynamics of mosquitoes in Juiz de Fora, a simplified domain is considered as shown in Fig. 2b. In the mathematical model we assumed homogeneous blocks by considering *ψ*, which represents the coefficient for mosquito area support. In both map and model blocks are separated by streets as shown in Fig. 2a, b and Additional file 1: Figure S1. The model parameters for blocks and streets are presented in Table 3. The component of the wind velocity in the *X* direction is assumed to be larger in both streets and park as compared to the wind velocity in the *Y* direction.

**Table 3:** Common dimensional parameter values used for Examples 2 and 3 (Juiz de Fora)

Parameter	Blocks	Streets	Park
*ψ*	1	0.3	0.8
*ν*_*x*_	0	-1.0 × 10^-1^	-5.0 × 10^-2^
*ν*_*y*_	0	5 × 10^-2^	2.5 × 10^-2^
*D*_*x*_	3.75 × 10^-3^	1.25 × 10^-2^	1.25 × 10^-2^
*D*_*y*_	3.75 × 10^-3^	1.25 × 10^-2^	1.25 × 10^-2^

**Fig. 9:**
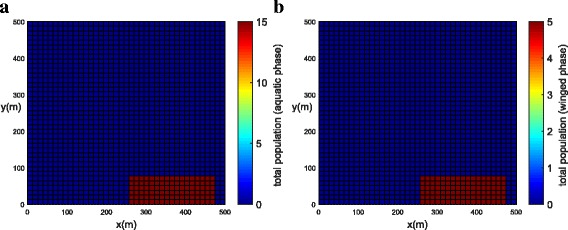
Example 2: initial condition for both populations in Juiz de Fora (*t* = 0). **a** Aquatic phase. **b** Winged phase

**Fig. 10:**
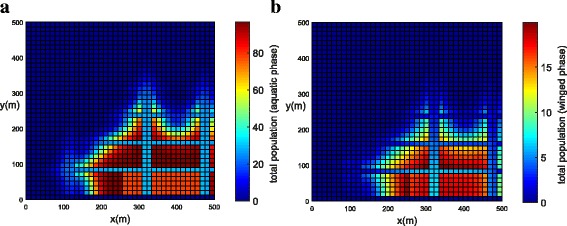
Example 2: population propagation in Juiz de Fora (*t* = 21 days). **a** Total population for aquatic phase (*ψ × A*). **b** Total population for winged phase (*ψ × M* )

**Fig. 11:**
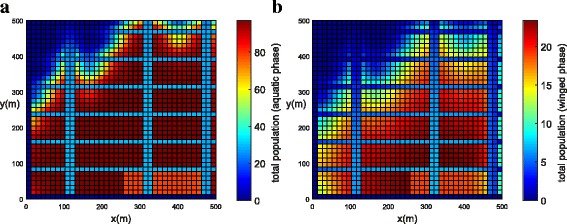
Example 2: population propagation in Juiz de Fora (*t* = 42 days). **a** Total population for aquatic phase (*ψ × A*). **b** Total population for winged phase (*ψ × M* )

In order to represent a higher concentration of mosquitoes inside the park and in the blocks, a higher value of (*ψ*) was chosen (Table 3). The initial populations for both phases have been concentrated in the park (Fig. 9a, b). The population concentration for both phases (winged and aquatic) is shown in Fig. 10a, b (*t* = 21 days) and Fig. 11a, b (*t* = 42 days). One observes that the population in both phases slowly diffuses from the park into the streets and blocks as shown in Fig. 10a, b. The population for both phases spread rapidly in the streets due to the presence of wind and larger value of diffusion coefficient when compared to the blocks and park. The mosquito population for both phases spread faster along the *X* direction due to the larger component of wind velocity along the *X* direction. As time progresses, the population of mosquitoes increase inside the blocks as expected (Fig. 11a, b). Notice that the invasion process is slower in the park as compared to the blocks. Furthermore, it is observed that the invasion process is much higher for the aquatic phase as compared to the winged phase near to the boundary (Fig. 11a, b). This may be due to the net influx for the winged phase being zero at the boundary of the domain.

### Example 3: City center of Juiz de Fora with chemical insecticides

In this section, the population dynamics of *Ae. aegypti* mosquitoes was studied in the same neighborhood of Juiz de Fora as in the previous example, taking into account the use of chemical insecticides. The model parameters are the same as in Example 2 (see Table 3) except for one parameter (*h*_*1*_). Note that for both simulations we used the same amount of insecticides.

#### City center of Juiz de Fora with insecticides along one street

As shown in Fig. 4a, one street with the surrounding area (grey color) was selected for the use of insecticides to control the population of mosquitoes. The value of parameter *h*_*1*_ is given in Table 4.

**Table 4:** Dimensional parameter values used for Example 3 (chemical insecticides used along one street)

Parameter	Blocks	Streets	Park
*h*_*1*_	0	7.6	0
*h*_*2*_	0	0	0

**Fig. 12:**
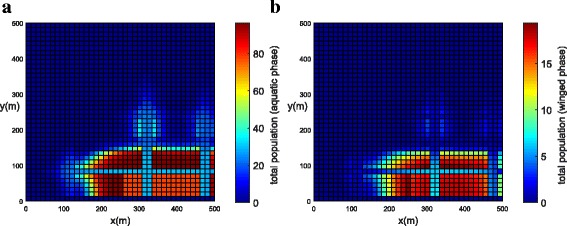
Example 3 (one street with chemical insecticides): population propagation in Juiz de Fora (*t* = 21 days). **a** Total population for aquatic phase (*ψ × A*). **b** Total population for winged phase (*ψ × M* )

**Fig. 13:**
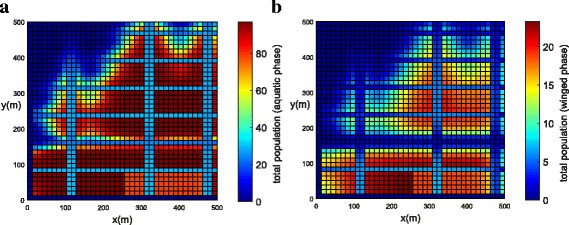
Example 3 (one street with chemical insecticides): population propagation in Juiz de Fora (*t* = 42 days). **a** Total population for aquatic phase (*ψ × A*). **b** Total population for winged phase (*ψ × M* )

The population concentration for both phases (winged and aquatic) is shown in Fig. 12a, b (*t* = 21 days) and Fig. 13a, b (*t* = 42 days). Initially, the population of mosquitoes was concentrated in the park. It was observed that the population for both phases slowly diffused from the park into the streets. Notice that, after the use of insecticides along a street, the mosquito concentration near the street was reduced (Fig. 13b).

The reduction in population for the winged phase is bigger when compared to the one for the aquatic phase. This happens because the killing term (*h*_*1*_) was used to control the mosquito population for the winged phase. However, the reduction in the winged phase has a direct impact on the reduction of the aquatic phase due to the dependence on the source term in Eqs. (1) and (2) on both *M*(*x*, *y*, *t*) and *A*(*x*, *y*, *t*).

#### City center of Juiz de Fora with insecticides along all streets

The effect of insecticides on the mosquito population was studied along all the streets of the city center in Juiz de Fora (Fig. 4b). The value of parameter *h*_*1*_ is given in the Table 5.

**Table 5:** Dimensional parameter values used for Example 3 (chemical insecticides used along all streets)

Parameter	Blocks	Streets	Park
*h*_*1*_	0	0.928	0
*h*_*2*_	0	0	0

**Fig. 14:**
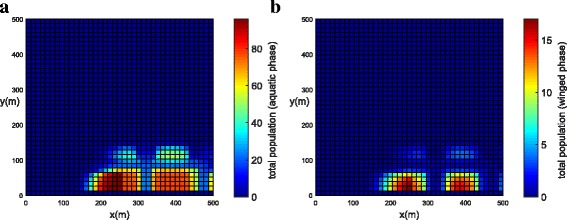
Example 3 (all streets with chemical insecticides): population propagation in Juiz de Fora (*t =* 21 days). **a** Total population for aquatic phase (*ψ × A*). **b** Total population for winged phase (*ψ × M* )

**Fig. 15:**
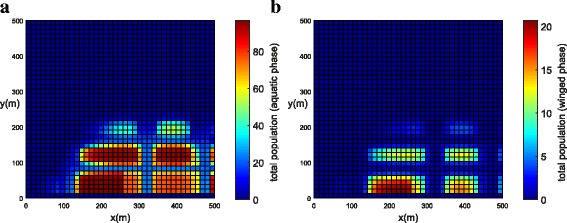
Example 3 (all streets with chemical insecticides): population propagation in Juiz de Fora (*t =* 42 days). **a** Total population for aquatic phase (*ψ × A*). **b** Total population for winged phase (*ψ × M* )

The population concentration for both phases (winged and aquatic) is shown in Fig. 14a, b (*t* = 21 days) and Fig. 15a, b (*t* = 42 days). Notice that, in this example when insecticides have been used in all streets, the mosquito concentration near the streets has been reduced significantly (Fig. 15a, b).

**Fig. 16:**
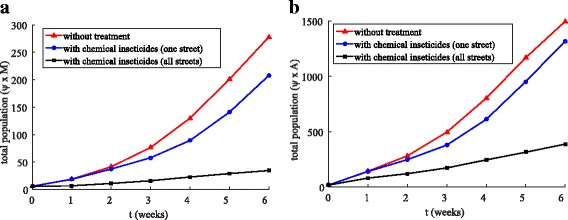
Comparison between Examples 2 and 3, where the red line represents the total population without chemical insecticides, the blue line represents chemical insecticides along a street and the black line represents chemical insecticides along all streets. **a** Total population for winged phase (*ψ × M* ). **b** Total population for aquatic phase (*ψ × A*)

Figure 16a, b shows the development of the total population of the mosquitoes for both phases calculated by integrating inside the domain: ∫_Ω_*ψM d*Ω and ∫_Ω_*ψAd*Ω for winged and aquatic phases, respectively. From the simulation, we observed that when insecticides were used uniformly along all the streets, the total population of mosquitoes for both phases was reduced significantly (Fig. 16a). When the insecticides were used uniformly along all the streets the winged phase population was reduced by 87.56% and an aquatic phase population was reduced by 74.09% (Fig. 16a). When the insecticides were used in greater concentrations, but only in one street, the winged population was reduced by 25.23% and an aquatic phase population was reduced by 11.92% (Fig. 16b).

It is clear from the simulation, that the chemical insecticides spread more uniformly along all the streets have a significant effect on the mosquito population for both winged and aquatic phases.

### Example 4: Copacabana Beach, Rio de Janeiro

In this section, we present the numerical results for the population dynamics of *Ae. aegypti* mosquitoes in Copacabana Beach, Rio de Janeiro (Fig. 3a).

Figure 3b shows the computational domain for the simulation of the current case containing 12 blocks. In the satellite map, each block contains several houses and apartments. For simplicity, we assume that the data are homogeneous inside the blocks setting the function *ψ*, which represents the coefficient for mosquito area support. Blocks are separated by streets as shown in Fig. 3a, b and Additional file 2: Figure S2. The model parameters for blocks, streets and beach are presented in Table 6.

**Table 6:** Dimensional parameter values used for Example 4 (Copacabana Beach, Rio de Janeiro)

Parameter	Blocks	Streets	Beach
*ψ*	1	0.3	0.8
*D*_*x*_	3.75 × 10^-3^	1.25 × 10^-2^	1.25 × 10^-2^
*D*_*y*_	3.75 × 10^-3^	1.25 × 10^-2^	1.25 × 10^-2^

During the daytime, the wind flows from the sea to the city; during the night, it follows the opposite direction. This periodic nature of the wind was incorporated in the simulation by introducing the variables *t*_*1*_ and *t*_*2*_, which indicate when this change occurs (see Table 7). The wind velocity near the beach is assumed to be twice as large than the velocity in the streets. The component of the wind velocity in the *X* direction is assumed to be larger in both the beach and streets as compared to the wind velocity in the *Y* direction. The wind velocity for blocks, streets and beach is presented in Table 7.

**Table 7:** The values used in wind velocity: inside the blocks, on the street and the beach

Parameter	Description	Blocks	Streets	Beach
*ν*_*x*_ (*t*_*1*_)	Wind velocity along the *X* direction during day time	0	-0.2	-0.4
*ν*_*y*_ (*t*_*1*_)	Wind velocity along the *Y* direction during day time	0	0.15	0.3
*ν*_*x*_ (*t*_*2*_)	Wind velocity along the *X* direction during night time	0	0	0
*ν*_*y*_ (*t*_*2*_)	Wind velocity along the *Y* direction during night time	0	-0.25	-0.5

**Fig. 17:**
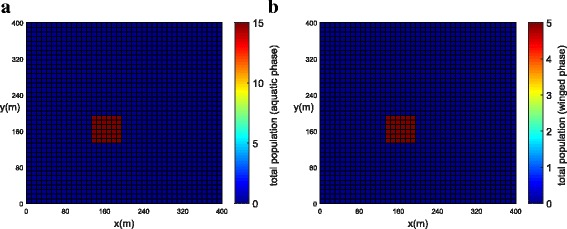
Example 4: initial condition for both populations in Copacabana Beach (*t* = 0). **a** Aquatic phase. **b** Winged phase

**Fig. 18:**
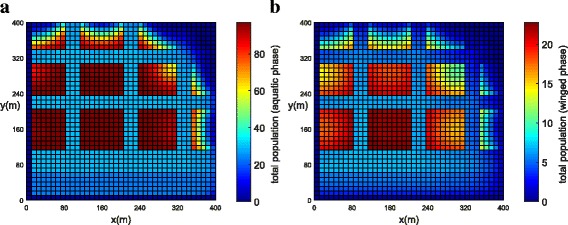
Example 4: population propagation in Copacabana Beach (*t* = 21 days). **a** Total population for aquatic phase (*ψ × A*). **b** Total population for winged phase (*ψ × M* )

**Fig. 19:**
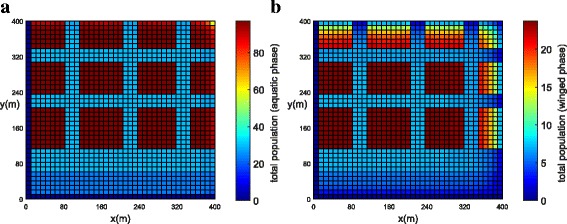
Example 4: population propagation in Copacabana Beach (*t* = 42 days). **a** Total population for aquatic phase (*ψ × A*). **b** Total population for winged phase (*ψ × M* )

Figure 17a, b shows the initial conditions for both winged and aquatic phases with the initial mosquito population concentrated in the small park. The population for both phases after *t* = 21 days is shown in Fig. 18a, b. We observed that, due to the greater wind velocity, the population of mosquitoes is lower near the beach. The population for both phases slowly diffused from the park into the streets and blocks as shown in Fig. 18a, b. The population for both phases spread rapidly in the streets due to the presence of wind and the higher value of the diffusion coefficient. We observed that the mosquito population in both phases spread quickly along the *X* direction due to the larger component of wind velocity along the *X* direction. As time progresses (*t* = 42 days), we observed that the population of mosquitoes increased inside the blocks (Fig. 19a, b). Furthermore, it was observed that the invasion process is higher for aquatic phase as compared to winged phase near the boundary due to the net influx for the winged phase being zero at the boundary of the domain (Fig. 19a, b).

## Discussion

In the present work, the model addressing the proliferation of *Aedes aegypti* mosquitoes was proposed. As far as we know, it is the most complete and realistic model for this issue. The two-dimensional model allowed us to take into account topography (building blocks, streets, beach areas, parks, forests, etc.), climate conditions (wind, temperature, humidity) and human action (insecticides). The modeling was based on the idea that the mosquitoes’ erratic movement depends significantly on the environment and needs to be taken into account when planning public health policies. The model depends on several parameters that can be measured or obtained through history matching. Despite other improvements, the presented model introduces the concept of mosquito area support, which allows the modeling of the non-uniformity of the mosquito population in a different environment.

In order to illustrate the model, four examples were presented for the population dynamics of *Ae. aegypti* mosquitoes for both the winged and aquatic phase at an urban scale.

The first example simulated the generic building blocks neighborhood. The mosquito population grew and dispersed rapidly due to the wind influence. In the second, the example city center of Juiz de Fora was simulated, showing the population growth spread from the park area into the building blocks. The third example used the same data as the previous one with the use of chemical insecticides attacking winged phase in two different configurations maintaining the same total amount of the insecticide. Furthermore, we observed that if the chemical insecticides spread more uniformly along all the streets in a neighborhood of a city, the insecticides have a significant effect on the mosquito population for both winged and aquatic phases. This observation gives a new insight regarding the optimal use of insecticides in a city. The fourth example describes Copacabana Beach area in Rio de Janeiro city taking into account strong and direction-changing winds.

## Conclusions

The presented methodology is a tool that can be employed to evaluate and to understand not only general epidemic risks but point city regions that are more susceptible to vector transmitted diseases. Moreover, as shown in the Example 3, the model can be used to increase efficiency of the existing mosquito population control techniques and test new methods theoretically, before involving the human population. Furthermore, this model can be coupled with SIR type models to investigate the disease dynamics in a city and include financial costs to assist in public health decisions.

## Additional files

Additional file 1: **Figure S1.** An enlarged view of the center of Juiz de Fora and its surroundings. The figure shows the surroundings of the city of Juiz de Fora (Source: Google Maps). The area marked on the map (red) is shown in Fig. 2a. (DOCX 1963 kb)

Additional file 2: **Figure S2.** An enlarged view of Copacabana in Rio de Janeiro and its surroundings. The figure shows the surroundings of the Copacabana Beach, Rio de Janeiro (Source: Google Maps). The area marked on the map (red) is shown in Fig. 3a. (DOCX 656 kb)

## Abbreviations

WHO: World Health Organization
FADEPE: Fundação de Apoio e Desenvolvimento ao Ensino, Pesquisa e Extensão
FAPEMIG: Fundação de Amparo à Pesquisa do Estado de Minas Gerais
FVM: Finite volume method
ODE: Ordinary differential equation
PDE: Partial differential equation
SIT: Sterile insect technique
