# Supplementary material for: Numerical modeling of mosquito population dynamics of Aedes aegypti
Source: Parasit Vectors. 2018 Apr 16;11:245. doi: 10.1186/s13071-018-2829-1 (PMC5902854; doi:10.1186/s13071-018-2829-1)

**Additional file 1: Figure S1. An enlarged view of the center of Juiz de Fora and its surroundings**

The figure shows the surroundings of the city of Juiz de Fora (Google Maps image). The area marked on the map (red) is shown in Fig. [2(a)](#_bookmark2).


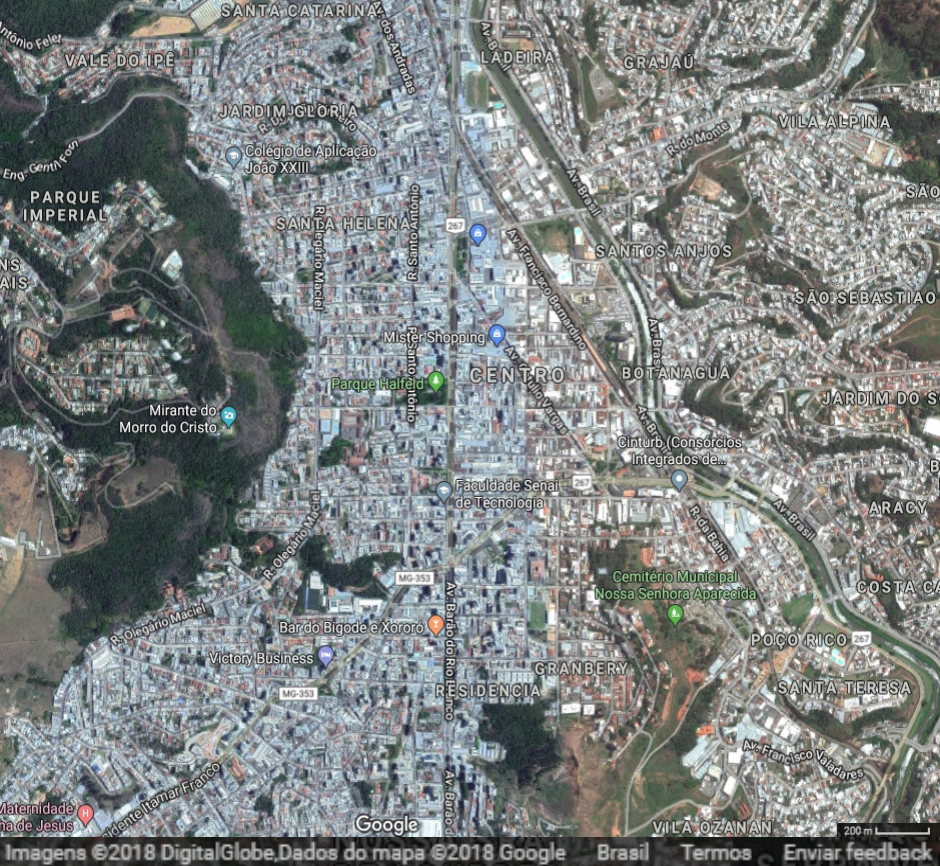

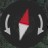

Supplement: Supplementary file 1 — Figure S1. An enlarged view of the center of Juiz de Fora and its surroundings. The figure shows the surroundings of the city of Juiz de Fora (Source: Google Maps). The area marked on the map (red) is shown in Fig. 2a. (DOCX 1963 kb) [file 13071_2018_2829_MOESM1_ESM.docx]
