# Supplementary material for: Numerical modeling of mosquito population dynamics of Aedes aegypti
Source: Parasit Vectors. 2018 Apr 16;11:245. doi: 10.1186/s13071-018-2829-1 (PMC5902854; doi:10.1186/s13071-018-2829-1)

**Additional file 2: Figure S2. An enlarged view of Copacabana in Rio de Janeiro and its surroundings**

The figure shows the surroundings of the Copacabana beach, Rio de Janeiro (Google Maps image). The area marked on the map (red) is shown in Fig. [3(a)](#_bookmark5).


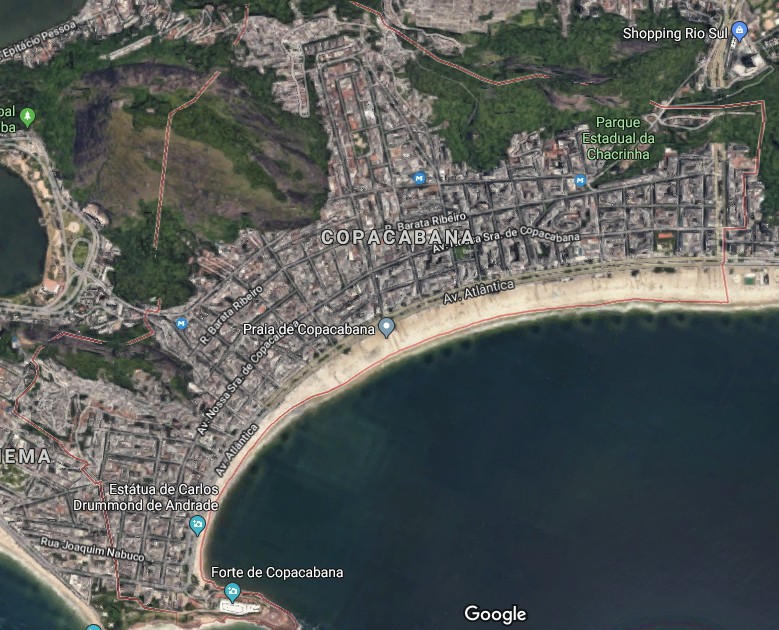

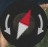

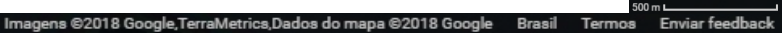

Supplement: Supplementary file 2 — Figure S2. An enlarged view of Copacabana in Rio de Janeiro and its surroundings. The figure shows the surroundings of the Copacabana Beach, Rio de Janeiro (Source: Google Maps). The area marked on the map (red) is shown in Fig. 3a. (DOCX 656 kb) [file 13071_2018_2829_MOESM2_ESM.docx]
